# Text Message Intervention Designs to Promote Adherence to Antiretroviral Therapy (ART): A Meta-Analysis of Randomized Controlled Trials

**DOI:** 10.1371/journal.pone.0088166

**Published:** 2014-02-05

**Authors:** David J. Finitsis, Jennifer A. Pellowski, Blair T. Johnson

**Affiliations:** Department of Psychology, Center for Health, Intervention, & Prevention (CHIP), University of Connecticut, Storrs, Connecticut, United States of America; Imperial College London, United Kingdom

## Abstract

**Background:**

The efficacy of antiretroviral therapy depends on patient adherence to a daily medication regimen, yet many patients fail to adhere at high enough rates to maintain health and reduce the risk of transmitting HIV. Given the explosive global growth of cellular-mobile phone use, text-messaging interventions to promote adherence are especially appropriate. This meta-analysis synthesized available text messaging interventions to promote antiretroviral therapy adherence in people living with HIV.

**Methods:**

We performed Boolean searches of electronic databases, hand searches of recent year conference abstracts and reverse searches. Included studies (1) targeted antiretroviral therapy adherence in a sample of people living with HIV, (2) used a randomized-controlled trial design to examine a text messaging intervention, and (3) reported at least one adherence measurement or clinical outcome.

**Results:**

Eight studies, including 9 interventions, met inclusion criteria. Text-messaging interventions yielded significantly higher adherence than control conditions (OR = 1.39; 95% *CI* = 1.18, 1.64). Sensitivity analyses of intervention characteristics suggested that studies had larger effects when interventions (1) were sent less frequently than daily, (2) supported bidirectional communication, (3) included personalized message content, and (4) were matched to participants’ antiretroviral therapy dosing schedule. Interventions were also associated with improved viral load and/or CD4+ count (*k* = 3; OR = 1.56; 95% *CI* = 1.11, 2.20).

**Conclusions:**

Text-messaging can support antiretroviral therapy adherence. Researchers should consider the adoption of less frequent messaging interventions with content and timing that is individually tailored and designed to evoke a reply from the recipient. Future research is needed in order to determine how best to optimize efficacy.

## Introduction

The efficacy of antiretroviral therapy (ART) depends on patient adherence to a daily medication regimen, yet many patients fail to adhere at high enough rates to maintain health and reduce the risk of transmitting HIV [1], [2]. In one recent meta-analysis of 84 observational studies, nearly 40% of participants reported less than 90% adherence [3]. For the individual, poor ART adherence can lead to quicker progression of HIV to AIDS [4], [5] while increasing the potential for the development of ART-resistant viral strains [6]–[9]. Drug-resistant strains of HIV virus may then be spread from the individual to community level through sexual and injection drug use networks [10], [11]. Promoting adherence sufficient to achieve viral suppression helps to create optimal outcomes in both individual and public health.

The current standard of care in promoting adherence is patients’ contact with their health care providers. Although research on adherence behavior shows the importance of adherence counseling on biological outcomes (e.g., viral load, CD4+ levels) implementation costs prove increasingly onerous as healthcare providers continue to be pressured to do “more with less” both in developed countries [12] and in resource-limited settings [13]. A number of reviews support the efficacy of behavioral interventions to promote ART adherence [14]–[16], yet these interventions have diverse approaches and variable financial costs. In order to translate effectively, the practice community requires effective interventions that minimize the necessary financial burden to implement [17].

One promising approach is the use of electronic text-messaging to deliver behavioral interventions [18]. In the last decade, mobile-cellular phone ownership has seen marked growth throughout the world [19]. Presently, there are nearly as many mobile-cellular phone subscriptions as there are people in the world; in the developing world, there are 89 subscriptions for every 100 people [20]. In a recent systematic review, Horvath and colleagues (2012) found high quality evidence of efficacy in interventions using short weekly messages [21] and World Health Organization guidelines include a strong recommendation to consider text messaging “for promoting adherence to ART as part of a package of adherence interventions [22].” Text-messaging provides researchers with the flexibility of personalizing message content, promoting bidirectional communication, and pairing message timing to ART dosage schedules [23]–[26]. The fact that there are so many variations of intervention design in text-messaging trials makes it unclear what specific ingredients actively and successfully promote adherence. Moreover, there are numerous studies currently in early phases of development and implementation that rely on messaging strategies [27]. A quantitative comparison of text-message intervention designs to promote ART adherence is therefore timely.

## Methods

### Selection Criteria

Intervention trials reported in English were included if they (1) targeted ART adherence in a sample of people living with HIV (PLWH), (2) used a randomized-controlled trial to examine an electronic text messaging intervention, and (3) reported at least one adherence measurement (i.e., self-report, pill count, electronic drug monitoring device, pharmacy refill) or biological outcome (i.e., viral load, CD4+ count). Studies were excluded when reported data were insufficient to calculate effect sizes and contacted authors were unable to provide the necessary additional data. Listings of excluded studies with justification for exclusion are included as supplemental tables in Appendix S2 and S3.

### Data Collection

Relevant studies were located through multiple strategies. We conducted serial searches of PsycINFO, PubMed, CINAHL, and ProQuest Dissertations and Theses for the years 1990 through October 2013, using Boolean strategy with the following terms: (cellphone OR “cellular phone” OR “mobile phone” OR “text message” OR “simple message service” OR SMS OR pager OR “two-way electronic messaging system”) AND (HIV OR HIV+ OR HIV-positive OR “people living with HIV/AIDS” OR “human immunodeficiency virus positive” OR “human immunodeficiency virus-positive” OR PLWHA) AND (“highly active antiretroviral therapy” OR “antiretroviral therapy” OR ART OR HAART) AND (adherence OR “medication adherence” OR MNA OR “medication non-adherence”). Unpublished literature was sought both through the ProQuest electronic database described above and through reviewing abstracts from conferences with a focus on HIV and adherence over the past five years and contacting authors to request inclusion of their work in this synthesis. Finally, the reference sections of all relevant studies were searched for any additional relevant literature. The numbers of results returned by each electronic database are included in Appendix S1.

### Coding of Interventions

Two reviewers independently abstracted data from the studies using a standardized coding form, including general information (e.g., trial location, year of data collection), participant characteristics (e.g., age, gender, race/ethnicity, baseline lab values), design parameters (e.g., length of intervention, type of control condition), messaging intervention features (e.g., device type, messaging frequency, unidirectional vs. bidirectional), and outcomes (e.g., method and frequency of assessment for adherence). Across coded variables there was a 94% reviewer agreement rate; coding discrepancies were reconciled through discussion.

### Risk of Bias of Individual Studies

Description of included studies’ methodological quality is an important facet of the systematic review. We assessed for risk of bias within individual studies using Downs and Black’s methodological quality scale [35], a 27-item instrument that assesses five dimensions of research methodology: reporting bias, external validity, measurement bias, confounding (selection bias), and statistical power. Two independent raters showed substantial [36] strength of agreement in their use of the instrument (*kappa* = 0.633; 95%CI = 0.515, 0.751; *p*<0.001). We report each of the instrument’s five sub-scales separately in order to provide a clearer appraisal of individual methodological elements than may be gained by a summary score [37]. All elements of this research are reported in accordance with PRISMA guidelines [38].

### Analytic Approach

Trials reported different numbers and types of outcomes; most reported multiple adherence outcomes (e.g., self-report, viral load). To uphold the assumption of independence, effect sizes (ESs) were averaged across adherence measures. When trials reported multiple follow up assessments, the last follow up was used in order to assess the greatest persistence and sustainability of the intervention effect. There was variation in the operationalization of adherence in this sample. Some studies reported outcomes entirely as continuous measures (*k* = 3), some in purely dichotomous terms (*k* = 4) and one reported a combination of continuous and dichotomous outcomes. For analyses, ESs were estimated with the standardized mean difference (*d*) [28]; ESs and inverse variance weights for each outcome were also calculated [29]. Final meta-analytical tests of derived ESs were performed using SPSS version 20.0 [30]. For presentation purposes, ESs were converted to ORs using the *d*_Cox_ transformation [31]. ORs greater than 1 reflect improvement in adherence or biological outcome; those less than 1 reflect declination.

Weighted mean ESs were calculated to estimate overall difference between treatment and control groups [32] on adherence as well as on biological outcome variables (viral load and CD4+), as clinical impact is the desired endpoint of adherence. ESs were analyzed using random-effects assumptions and the magnitude of heterogeneity across ESs was assessed using the *I^2^* statistic [33], which is known to have low statistical power when the number of available studies is small [34]. There are now sufficient trials available to begin to evaluate the literature, to determine the consistency and magnitude of adherence gains, and to conduct sensitivity analyses that may provide clues as to the active ingredients necessary to create better adherence to ART. Based on recent qualitative and theoretical work [22]–[26], we expected *a priori* that messaging interventions would work better when provided more frequently using messaging that was individually tailored, offered an opportunity for bidirectional communication between parties, and were timed to correspond to the patient’s ART dosing schedule. Analyses using the *Q* statistic as a measure of variance in a meta-analytic analog to the one-way ANOVA [30] assessed whether intervention characteristics across between-group interventions explained variability in the ESs, presented as stratified sensitivity analyses.

## Results

**Figure 1:**
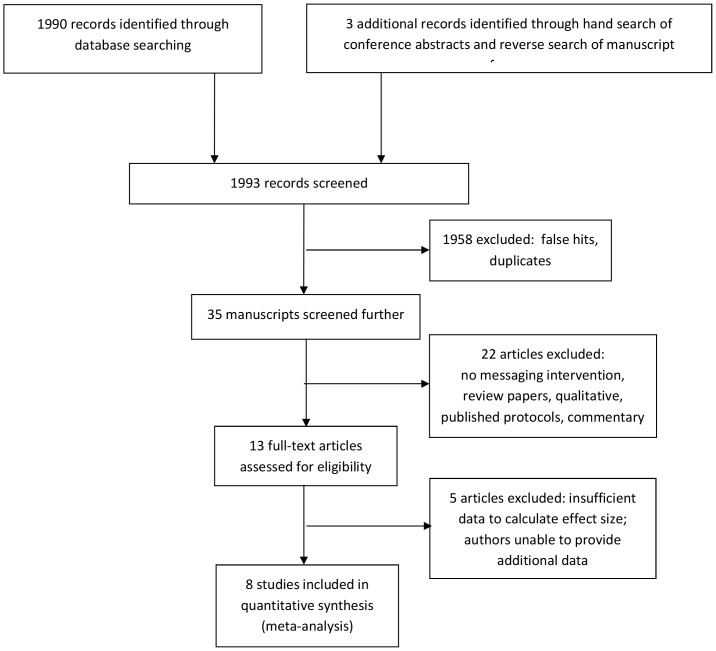
Literature search results.

Eight studies [39]–[46] reporting 9 interventions met inclusion criteria (see Figure 1); Table 1 summarizes their features. The sample consists of 7 peer-reviewed journal articles and an unpublished dissertation, all written in English. Half (*k* = 4) of included studies were conducted in North America, 3 took place in Africa, and 1 in South America. Most data (70%) were collected during or after the year 2007 (range = 1999–2010). In total, 1,785 individuals consented and 1,463 (82%) of these were retained to at least one follow-up time point. The sample was 49% female and African participants made up 65% of the sample. Mean participant age was 40.00 years (range = 36.21–42.95). In those trials that reported it, mean baseline CD4+ was 291.83 cells/µL (*SD* = 107.46; *k* = 5) and viral load was 4.34 log_10_ copies/mL (*SD* = 0.32; *k* = 4).

**Table 1:** Characteristics of included studies.

Author, Year, and Journal	Location	Sample Characteristics	Design	Measurement	Effect Size Information^a^	Methodological Quality Scores^b^	
da Costa et al., 2012, *International* *Journal of Medical Informatics*	Sao Paulo, Brazil	29 women; ethnicity: Latin-American;mean age: 35.6; taking first or secondline regimens with at least 3 monthsat VL <400 copies and CD4+ >200	150 days, with 3x/weekunidirectional standardizedSMS messaging vs. control(usual care)	SR, PC, EDM at baseline andmonthly follow-ups.Dichotomized at 95% for allmeasures	OR = 2.47 (0.42 to 14.25)	RB =	8
						EV =	1
						MB =	6
						SB =	4
						PW =	0
							
Hardy et al., 2011, *AIDS* *Patient Care and STDs*	Boston, MA, USA	23 men (53%) and women (47%);ethnicity: minority 63%, white 37%;mean age 43.0; at least 3 monthswithout changes to medicationregimen, reporting <85% adherencein the last 7 days	42 days, with daily bidirectionalpersonalized SMS messagingmatched to time of ART dosingvs. control group with beepermatched to time of ART dosing	SR, PC, EDM at baseline, 3,and 6 weeks. Reported ascontinuous variables	OR = 1.75 (0.39 to 7.86)	RB =	10
						EV =	1
						MB =	5
						SB =	4
						PW =	0
							
Lester et al., 2010**,** *Lancet*	Nairobi, Kenya	538 men (35%) and women (65%);ethnicity: African; mean age: 36.6;initiating ART for the first time	365 days, weekly bidirectionalstandardized SMS messaging vs.control (usual care)	SR, VL post only. Dichotomizedat 95% (SR) and <400 copies (VL)	OR = 1.53 (1.16 to 2.02)	RB =	11
						EV =	1
						MB =	6
						SB =	6
						PW =	1
							
Mbuagbaw et al., 2012 *PLoS One*	Yaounadé, Camaroon	200 men (26%) and women (74%);ethnicity: African; mean age: 40.2;on ART for at least one month	180 days, weekly bidirectionalstandardized SMS messaging vs.control (usual care)	SR(x2), PR, CD4+ at baseline,3, and 6 months. Dichotomized at 90%,95%, and 100% (SR)	OR = 1.41(0.76 to 2.71)	RB =	10
						EV =	2
						MB =	6
						SB =	6
						PW =	1
							
Musser, 2001 (Unpublished)	St. Louis, MS, USA	22 men (91%) and women (9%);ethnicity: 77% white 18% black,5% other; mean age: 44.6; with un-detectableVL/CD4+ >200	14 days, daily bidirectionaluniform SMS messaging vs.control (usual care)	SR at 2 weeks. Reported as acontinuous variable	OR = 2.03 (0.48 to 8.48)	RB =	9
						EV =	3
						MB =	4
						SB =	3
						PW =	0
							
Pop-Eleches et al., 2011**,** *AIDS*	Nyanza Province, Kenya	431 men(34%) and women(66%);ethnicity: African; mean age: 36.2;initiating ART within the last3 months	336 days, with daily^‡^ andweekly* unidirectional standardized SMSmessaging arms vs. control(usual care)	EDM at 3, 6, 9, and 12 months.Dichotomized at 90% adherence	OR* = 1.02 (0.52 to 2.02) OR‡ = 1.68(1.05 to 2.68)	RB =	10
						EV =	0
						MB =	5
						SB =	4
						PW =	1
							
Safren et al., 2003**,** *AIDS Care*	Boston, MA, USA	82 men (80%) and women (20%);ethnicity: 43% white, 30% black, 17%Hispanic, 10% other; mean age:NR; with 90% or lower adherenceafter 2 week EDM surveillance	84 days, with multiple dailyunidirectional text-based pagermessaging matched to timeof ART dosing vs. control(usual care)	EDM at 2 and 12 weeks.Reported as a continuousvariable	OR = 1.43 (0.64 to 3.18)	RB =	6
						EV =	1
						MB =	5
						SB =	4
						PW =	0
							
Simoni et al., 2009**,** *Journal of Acquired* *Immune Deficiency Syndromes*	Seattle, WA, USA	226 men (76%) and women (24%);ethnicity: 47% white, 30% black, 11%Hispanic, 12% other; mean age: 40; 62% ART naïve, 38% changingor restarting regimens	90 days, with multiple dailybidirectional text-based pagermessaging matched to timeof ART dosing vs. control (usualcare)	SR, EDM, VL, CD4+^c^ at baseline, 3, 6,and 9 months. Dichotomized at 100%(SR) and reported as continuousvariables	OR = 1.65 (0.89 to 3.07)	RB =	10
						EV =	0
						MB =	5
						SB =	6
						PW =	0
							

*Note.* SR = self-report questionnaire; PC = pill count; EDM = electronic drug monitoring; PR = pharmacy records; VL = viral load; CD4+ = T-cell count; NR = not reported. ^a^Averaged when there is more than one measure of adherence. ^b^ methodological quality sub-scales (range): RB =  reporting bias (0–11); EV =  external validity (0–3); MB =  measurement bias (0–7); SB =  sampling bias (0–6); PW =  power (0–5). ^c^ CD4+ at baseline and 3 months only.

### Design and Intervention Characteristics

The average length of intervention was about six months (*M* = 177.49 days; range = 14–365). The mean number of outcomes reported was 2.33 (range = 1–4). The majority of interventions (*k* = 5) used multiple adherence measurements while three studies relied on a single form of adherence measurement: self-report [41] and electronic drug monitoring (EDM) [43], [44]. Self-report and EDM were the most commonly used adherence measurements (*k* = 6), followed by biological measures (i.e., viral load, CD4+; *k* = 3). A minority of studies employed in-person pill counts (*k* = 2) or pharmacy refill data (*k* = 1) to operationalize adherence. Among the 6 (67%) interventions that dichotomized adherence, threshold values ranged from 80 to 100% (median = 95%).

Seven (78%) interventions used cellular-mobile phones [38]–[43] and two used two-way alphanumeric pagers [44], [45], with no statistically significant effect by device type observed (between groups *Q* = 0.56; *p* = 0.46). Messages were sent daily in 5 (56%) interventions. Message timing was matched to dosing schedule in 4 (44%) interventions. Three (33%) interventions personalized message content to the individual participant. Five (56%) interventions supported bidirectional communication in the design (i.e., participants were allowed, encouraged, or required to respond to incoming messages).

### Did Text Messaging Improve Adherence?

**Figure 2:**
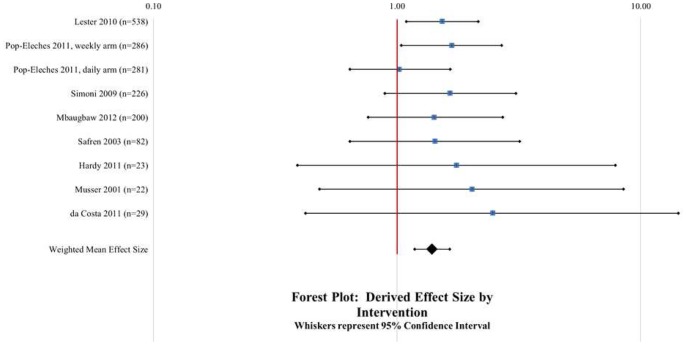
Forest plot of weighted mean effect size by study with aggregate mean effect size.

Table 2 shows the weighted mean ESs across the outcome measures. Overall, the interventions significantly improved the average adherence outcome (*k = *9; OR = 1.39; 95% *CI* = 1.18, 1.64). Table 2 also shows that mean effects on individual measures all fell within the confidence interval for the average effect; that is, the type of adherence measure appears not to have played a marked role in how large an effect appeared. Of note, biological outcomes also had a weighted mean effect size that achieved statistical significance (*k* = 3; OR = 1.56; 95% *CI* = 1.11, 2.20); individual outcome measures did not always achieve statistical significance, but it should be noted that the numbers of available studies limited statistical power. A forest plot depicting the distribution of individual study ESs, averaged across available adherence measures, appears in Figure 2. There was little indication of heterogeneity in the averaged effect sizes, and within individual measures there was only one case, CD4+ counts, that was significantly heterogeneous.

**Table 2:** Mean effect sizes by measurement method.

Measure	*k*	*OR* (95% *CI*)	*I* ^2^ (95% CI)
Self-report	6	1.48 (1.09, 2.01)	25.81 (0, 68.96)
Electronic drug monitoring	6	1.23 (0.97, 1.56)	3 (0, 59.01)
Viral load	2	1.52 (1.17, 1.95)	0 (0, 60.66)
CD4+	2	2.02 (0.75, 5.41)	84.45 (36.29, 96.21)
Pill count	2	1.70 (0.52, 5.59)	0 (0, 99.63)
Pharmacy refills	1	1.70 (0.52, 5.59)	–
Biological outcomes(CD4+ and viral load)	3	1.56 (1.11, 2.20)	47.20 (0.00, 84.52)
Mean adherence(all outcomes above,averaged)	9	1.39 (1.18, 1.64)	0.00 (0.00, 51.69)

*Note.* Mean effect sizes (OR) greater than 1 indicate improvement in the outcome of interest relative to the control arm. *k* = number of interventions.

In Table 1, we report individual study scores on the five sub-scales of methodological quality assessed by the Downs and Black scale. Individual studies showed low risk of reporting bias overall (range of possible scores = 0–11; *M* = 9.25; *SD* = 1.58). There was also low risk of measurement bias (range of possible scores = 0–7; *M* = 5.25; *SD* = 0.71) and sampling bias (range of possible scores = 0–6; *M* = 4.62; *SD* = 1.19) among individual studies. However, external validity was low in most studies (range of possible scores = 0–3; *M* = 1.12; *SD* = 0.99) and a substantial proportion of individual studies (62.5%; *k* = 5) were underpowered.

Given the small sample size, determining moderators of ES magnitude would typically seem ill-advised; yet, an important question to be answered through this meta-analysis involves determining the active ingredients of the interventions. In addition, several design variations were fairly evenly distributed across studies in this review. We therefore stratified analyses across those intervention design parameters that were reasonably well-distributed in the sample. The results of these sensitivity analyses appear in Table 3. Studies were more likely to report larger effects when messaging interventions (1) were not sent daily, (2) supported bidirectional communication, (3) included personalized message content, and (4) were matched to participants’ ART dosing schedule. Although none of these patterns achieved statistical significance as sub-group analyses, the mean effect sizes for these categories were statistically significant whereas the effect size sometimes did not achieve significance when these categories were absent.

**Table 3:** Mean effect sizes by Intervention Characteristics of Randomized Controlled Trials.

Intervention Characteristic	*OR* (95% *CI*)	*k*
Daily Messaging		
Yes	1.25 (0.46, 1.68)	5
No	1.46 (1.20, 1.79)	4
Bidirectional Communication		
Yes	1.57 (1.22, 2.01)	5
No	1.26 (1.00, 1.58)	4
Personalized Message Content		
Yes	1.69 (1.03, 2.77)	3
No	1.36 (1.14, 1.62)	6
Messages Matched to Dose Schedule		
Yes	1.72 (1.08, 2.75)	4
No	1.35 (1.13, 1.61)	5

*Note.* Odds ratios (OR) gauge the success of the interventions at increasing adherence as represented by its average across available measures for each study, where larger values indicate better success. Each moderator listed was evaluated individually without controlling for other listed moderators; that is, analyses are bivariate.

## Discussion

The main result of this meta-analysis is that text-messaging interventions improve HIV treatment outcomes. In this sample, compared to control groups, those receiving text messages’ support were more likely to maintain adherence thresholds at follow up and meet the clinical goals of lower viral load and higher CD4+ count (see Table 2). In addition, sensitivity analyses suggest that several design elements can enhance intervention effects further. Specifically, interventions messaging participants once or more times daily demonstrated smaller effects than interventions that messaged several times a week or weekly (Table 3). This apparent relationship between increased frequency and decreased response was contrary to our predictions but may have resulted from habituation, response fatigue and the possible intrusion that multiple daily messaging could represent. At the same time, it is not clear if the optimal “dosage” is somewhere between daily and weekly messaging or if a less than weekly messaging frequency would show the largest effects.

Another design element in need of further exploration is that of directionality. Designs that allowed, encouraged, or required message recipients’ response exhibited better outcomes than “one-way” reminder messages, which may have occurred because of enhanced engagement. Engagement is the “tuning in” of the recipient to the communication and has been identified as a necessary prerequisite for health behavior change [47]. Greater engagement of the recipient with the message may take place when the recipient acknowledges or replies to the sender. Another possible factor is the extent to which bidirectional communication bolsters the patient-provider relationship, increasing trust [11]. Evidence suggests that patients are better able to manage their care and are more likely to achieve adherence goals when they perceive their providers care about and like them [48], [49]. It is possible that messaging back and forth with clinic staff sufficiently evokes a relationship dynamic as to further bolster the extant patient-provider relationship.

A third element of messaging design investigated here was that of individual tailoring of message content. Interventions that used participant input to generate personalized message content saw larger effects on outcomes than those that sent uniform messages to all participants. While providers are certainly the experts of disease, patients are the experts of themselves. Interventionists who sought the participants’ own words and preferences may have produced intervention content that was less intrusive and more relatable for participants.

The final facet of intervention design considered in this review is messaging that is matched to dosage timing. A greater effect was observed in studies where messaging corresponded to the time of an ART dose, an effect that seems akin to that of receiving a timed reminder [18]. Although cellular phones typically have a built-in function allowing users to set multiple alarms, messaging paired to ART dosage lowers this barrier even further. Still, it is unclear how adoption of this element would correspond or conflict with the adoption of a less frequent messaging schedule overall and which element promotes the largest effect. These are all important areas for future research to explore.

There are several limitations of the current study. Although inclusion criteria were kept as broad as possible while maintaining focus on the research question, only 8 studies representing 9 interventions met inclusion criteria. Although 5 additional reports (see Appendix S2) met criteria for initial inclusion, reporting was insufficient for data abstraction (e.g., messaging interventions were bundled together with other co-interventions; only a conference abstract was available). Although we considered including these studies in the sample, we ultimately omitted them as too ambiguous. Whereas authors were contacted, none were able to provide additional data to allow inclusion of their work in this review. This small sample limitation raises a specter of publication bias and increases the importance of assessing for bias within individual studies. Yet, only 2 (22%) of the 9 intervention ESs reported herein are significant (Figure 2), which is not strongly suggestive of publication bias in itself given that all but one of the studies were subjected to peer-review. Using a validated methodological quality assessment tool [35] we concluded that there was low overall risk of bias in individual studies. Nonetheless, there were discrete areas of methodological weakness that do contribute limitations to the present study. External validity scores were low in this sample of studies as few studies used random sampling of a representative frame in their design. Moreover, most studies lacked sufficient statistical power to detect intervention effects. The developmental trajectory of intervention research is to first establish the efficacy of an intervention under internally valid conditions before expanding research to assess its effectiveness in a more ecologically valid setting. We felt that in the context of a burgeoning mHealth adherence intervention literature it was important to include all available studies in order to sample and compare the greatest diversity of intervention designs.

The sampling frame is another important contextualizing factor through which to view these findings. The majority of intervention trials sampled an ART-naïve HIV+ population and only 2 interventions targeted individuals with demonstrated non-adherence. It is unclear what effect a predominantly ART naïve sample may have had on the overall effect size estimates. If baseline adherence behavior approaches a normal distribution in this population, a “signal-noise” effect may occur with inherently adherent individuals in the intervention group attenuating the perceived effect, which has been observed elsewhere [14]. Future research comparing the effects of messaging interventions in samples of ART naïve and ART “experienced” (i.e., those with documented nonadherence) PLWH can serve the process of translational research and the formulation of best practices.

Although the effect size associated with text-messaging is of fairly modest size, one should recognize that many of the control groups in the sampled trials received standard of care interventions, which logically would enhance adherence and thereby decrease the effect size in the treatment vs. control comparisons that the current meta-analysis examined [50]. Future meta-analyses could capture this variance if change over time were examined instead of the between-groups comparison, and such an analysis would no doubt reveal a much larger effect size for text messaging in increasing adherence to highly active antiretroviral therapy.

Variation in the operationalization and measurement of adherence has been described before in the adherence literature [14], [51] and remains a limitation of the present study. While means of assessing adherence have proliferated, a true “gold standard” of adherence measurement remains elusive with each method possessing both strengths and limitations [52], [53]. Comparisons among different measurement strategies typically yield significantly correlated but not identical results [54]–[59]. In this review, the number and types of measurement varied across included studies; effects by individual outcome measure also varied and did not always achieve statistical significance. It is noteworthy that the numbers of available studies limited statistical power, particularly at this level of analysis. While we feel that aggregating differently derived effect sizes in each study is the most appropriate approach in this case, it is nonetheless a limitation of this study that the limited statistical power of the small sample size prevented a more detailed analysis of effect size by adherence measure.

The implementation of adherence thresholds also varied in our sample. The task of operationalizing “sufficient” adherence is necessarily fraught with multiple contributing factors. Providers historically have set a goal of perfect (100%) adherence [60], however, changes in drug potency appear be moving the goal posts. Evidence is accumulating that newer, more potent combination therapies are effective at imperfect (i.e., less than 95%) adherence levels [61], [62]. Further, Lima and colleagues reported that duration of viral suppression may moderate risk of viral rebound secondary to an episode of nonadherence with a longer duration lowering risk [63]. These findings suggest that less than 100% adherence can still be effective, but leave the operationalization of adherence a somewhat open question. The variation present in the small sample of studies included in this review makes estimation across studies more difficult.

Finally, this review combined results from first-wave large scale trials with early pilot studies, resulting in a wide range of intervention and follow up durations (Table 1). We attempted to mitigate this by considering the last follow up time points within each study. While it is a limitation of this review that more reports of longer duration interventions with follow up were not available, we felt the inclusion of smaller scale, shorter duration trials was important and necessary in order to address the research questions. Moreover, a recently published individual patient data meta-analysis of large scale (*k* = 3) trials [40], [42], [43] found similar overall effects of intervention over control conditions [64]; this suggests that our inclusion of pilot studies may not have unduly biased our overall findings.

Electronic messaging interventions can help PLWH achieve enhanced adherence to ART and improved clinical outcomes. The simple fact that such interventions can be disseminated *en masse* using technology that even in the developing world is increasingly mainstream makes it exceedingly cost-effective to implement; this is in addition to the savings in medical cost offset secondary to increased ART adherence. All of this bodes well for the eventual translation of this intervention strategy from research into practice. Future research is needed to model the impact of this type of intervention on health care systems. A formal cost-effectiveness analysis can compare the costs to implement electronic messaging with the benefits of enhanced ART adherence upon (1) drug resistance and the attendant escalation of pharmacotherapy; (2) the number of HIV-related hospitalizations/care; and (3) HIV transmission rates.

In conclusion, there is good reason for optimism about text-messaging interventions to promote ART adherence. Researchers should consider the adoption of a less than daily frequency of messaging that is individually timed and tailored and designed to evoke a reply from the recipient. Future research is needed to formally compare these design and intervention characteristics described and analyzed here in order to titrate each of these parameters to an optimal effect.

## Supporting Information

Appendix S1 **Literature Search Results by Database.**
(DOCX)

Appendix S2 **Reports excluded after full-text review with justification.**
(DOCX)

Appendix S3 **Reports excluded based on title and abstract with justification.**
(DOCX)

Checklist S1 **PRISMA reporting checklist.**
(DOC)
