# Supplementary material for: Text Message Intervention Designs to Promote Adherence to Antiretroviral Therapy (ART): A Meta-Analysis of Randomized Controlled Trials
Source: PLoS One. 2014 Feb 5;9(2):e88166. doi: 10.1371/journal.pone.0088166 (PMC3914915; doi:10.1371/journal.pone.0088166)
Supplement: Appendix S1 — Literature Search Results by Database. (DOCX) [file pone.0088166.s001.docx]

Appendix S1. Search results by database

| Database | Results returned |
| --- | --- |
| PubMed/Medline | 46 |
| PsycINFO | 19 |
| CINAHL Plus with Full Text | 14 |
| Proquest Dissertations & Theses | 1901 |
| Total | 1990 |
