# Supplementary material for: Text Message Intervention Designs to Promote Adherence to Antiretroviral Therapy (ART): A Meta-Analysis of Randomized Controlled Trials
Source: PLoS One. 2014 Feb 5;9(2):e88166. doi: 10.1371/journal.pone.0088166 (PMC3914915; doi:10.1371/journal.pone.0088166)
Supplement: Appendix S2 — Reports excluded after full-text review with justification. (DOCX) [file pone.0088166.s002.docx]

Appendix S2. Reports excluded after full-text review with justification

| First author and year | Source | | Justification for exclusion |
| --- | --- | --- | --- |
| Ingersoll (2012) | IAPAC (abstract 211) | Poster: authors unable to provide additional data for inclusion | |
| Rodriques (2012) | PLOS ONE | Not an RCT | |
| Fairley (2003) | Intl J STD AIDS | Cointerventions; author unable to provide additional data for inclusion. | |
| Curioso | University of Washington | Dissertation abstract: author unable to provide additional data for inclusion | |
| Ikeda (2012) | IAC 2012 | Poster: authors unable to provide additional data for inclusion | |
|  |  |  | |
