# Supplementary material for: Text Message Intervention Designs to Promote Adherence to Antiretroviral Therapy (ART): A Meta-Analysis of Randomized Controlled Trials
Source: PLoS One. 2014 Feb 5;9(2):e88166. doi: 10.1371/journal.pone.0088166 (PMC3914915; doi:10.1371/journal.pone.0088166)
Supplement: Appendix S3 — Reports excluded based on title and abstract with justification. (DOCX) [file pone.0088166.s003.docx]

Appendix S3: Reports excluded based on title and abstract with justification

| First author and year | Source | Justification for exclusion |
| --- | --- | --- |
| Andrade (2004) | International Conference on AIDS abstract register | No control group |
| Bamberger (2000) | American Journal of Public Health | No intervention |
| Barnighausen (2011) | The Lancet infectious diseases | Review |
| Chi (2010) | The Lancet | Commentary |
| Coomes (2012) | AIDS Care | Review |
| Crankshaw (2010) | AIDS Patient Care & STDs | No control group |
| Curioso (2009) | AMIA ...Annual Symposium proceedings | No intervention |
| De Costa (2010) | BMC Medical Research Methodology | Protocol report |
| Dunbar (2003) | Journal of the American Medical Informatics Association | No intervention |
| Haberer (2010) | AIDS and Behavior | No control group |
| Harris (2010) | Telemedicine journal and e-health | No control group |
| Horvath (2012) | Cochrane Database of Systematic Reviews | Review |
| Kalichman (2011) | AIDS Patient Care and STDs | No text messaging intervention |
| Kurtyka (2008) | Unpublished Dissertation | No text messaging intervention |
|  |  |  |
| Lester (2009) | Trials | Protocol report |
| Maqutu (2011) | AIDS Care | No intervention |
| Mbuagbaw (2011) | Trials | Protocol report |
| Puccio (2006) | AIDS Patient Care and STDs | No control group |
| Roux (2011) | Journal of acquired immune deficiency syndromes | No intervention |
| Shet (2010) | AIDS and Behavior | Protocol report |
| Sidney (2011) | AIDS Care | No adherence outcomes |
| Yard (2011) | AIDS and Behavior | Secondary analysis of included study (Simoni, 2009) |
